# Extraneural metastases from cranial meningioma: a case report

**DOI:** 10.1186/1748-717X-4-20

**Published:** 2009-07-06

**Authors:** Mirna Abboud, George Haddad, Mireille Kattar, Ibrahim Aburiziq, Fady B Geara

**Affiliations:** 1 Department of radiation Oncology, The American University of Beirut Medical Center, Bliss Street, Beirut, Lebanon; 2 Division of Neurosurgery, the American University of Beirut Medical Center, Bliss Street, Beirut, Lebanon; 3 Department of Pathology, the American University of Beirut Medical Center, Bliss Street, Beirut, Lebanon

## Abstract

Extracranial metastases from brain meningiomas is a rare, but well-documented entity. Metastases occur mostly in the lungs, pleura and liver, but may also affect lymph nodes and bones. We report here on a patient who was treated for an atypical brain meningioma with multiple surgeries and multiple sessions of stereotactic radiosurgery with good control of his brain disease. Thirteen years after diagnosis, he developed bilateral large sacroiliac and abdominal metastases.

## Introduction

Meningioma is a common intracranial tumor. Most meningiomas are benign slowly growing tumors that histologically correspond to World Health Organization (WHO) grade I. However, atypical (WHO grade II) and anaplastic (WHO grade III) meningiomas show a more aggressive biological behavior with a high risk of local recurrence and a less favorable prognosis. Extracranial dissemination of meningiomas has been rarely reported. We present here an unusual case of a patient who was treated for an atypical brain meningioma originally diagnosed in 1994 and treated by multiple surgical resections, several courses of stereotactic radiosurgery and radiotherapy, and a course of whole-brain radiotherapy with good control of his cranial disease. His disease remained confined to the brain for thirteen years, after which he developed bilateral large sacroiliac and abdominal metastases.

## Case presentation

The patient is a 45-year-old man who was admitted for the first time in 1994 at the age of 31 for persistent headache. Work-up revealed a large left tentorial meningioma. He had no history of cranial radiation during childhood or clinical features of neurofibromatosis type I or II. He underwent gross total surgical resection. Pathologic evaluation, which was recently re-reviewed, showed an atypical meningioma with the following features: hypercellularity with sheets of monotonous meningothelial cells displaying a prominent mitotic activity with around 4 mitoses per 10 high power fields. In May of 1997, follow-up brain MRI revealed a recurrence in the vicinity of the original tumor bed. Surgery was performed with gross total tumor removal. Pathologic evaluation again showed meningioma with similar pathologic features. In September 1999, he developed multiple small tumor recurrences in the parietal, occipital and cerebellar lobes for which he underwent stereotactic radiosurgery (SRS) with excellent results on tumor control. In September 2001, the patient developed additional recurrent lesions in multiple sites in the infratentorial, retroclival, left optic nerve sheet, and foramen magnum regions.

**Figure 1:**
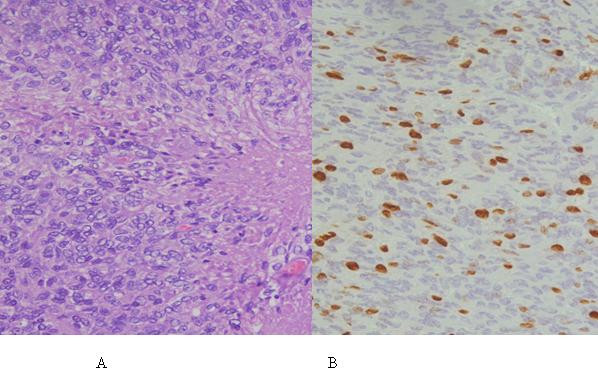
**(A) H&E stain on the intracranial specimen resected in 2007 showing high cellularity and sheets of meningothelial cells with a mitotic activity of 4 per 10 high power fields with foci of necrosis (center and lower right)**. (B) Ki-67 immunostaining with the MIB-1 antibody showing a labeling index of around 15%.

He was again treated by stereotactic radiosurgery with good tumor control defined as a complete disappearance or progressive decrease in lesion size. In June 2003, he received further SRS treatment to new cerebellar, premedullary and craniocervical junction lesions. In February 2004, he developed numerous lesions outside previously treated sites and was given whole brain radiation therapy (WBRT) of 36 Gy as a palliative therapy because of the multiplicity and the progression of the brain lesions. Surprisingly many of the small lesions disappeared after WBRT and the patient remained asymptomatic until August 2005 when he developed left facial numbness. Brain MR imaging at that time showed a recurrent lesion in the left cerebellopontine (CP) angle, an area that was previously treated by SRS and WBRT. He was given 10 sessions of stereotactic radiotherapy (30 Gy in 10 fractions) and achieved a good partial response but this lesion grew back and caused progressive left facial numbness due to left Vth nerve compression. This was treated by another craniotomy and surgical resection. Histopathologic examination showed meningioma with the same pathologic features and additional elements like the presence of foci of geographic necrosis and a hemangiopericytomatous pattern of vasculature (Figure 1A). Mitotic count was now up to five per ten high power fields and Ki-67 labeling index was up to 15% (Figure 1B). The tumor was focally positive for epithelial membrane antigen (EMA) and negative for CD31 and CD34. The lack of CD34 immunostaining excluded the possibility of hemangiopericytoma or solitary fibrous tumor of the meninges. Occasional cells were weakly positive for S-100 protein. In July 2007, follow-up MRI showed tumor progression at the same CP angle site for which a redo SRS treatment was debated and finally delivered without significant acute toxicity. Of note, the question of chemotherapy was raised many times but the patient refused to receive any systemic therapy.

**Figure 2:**
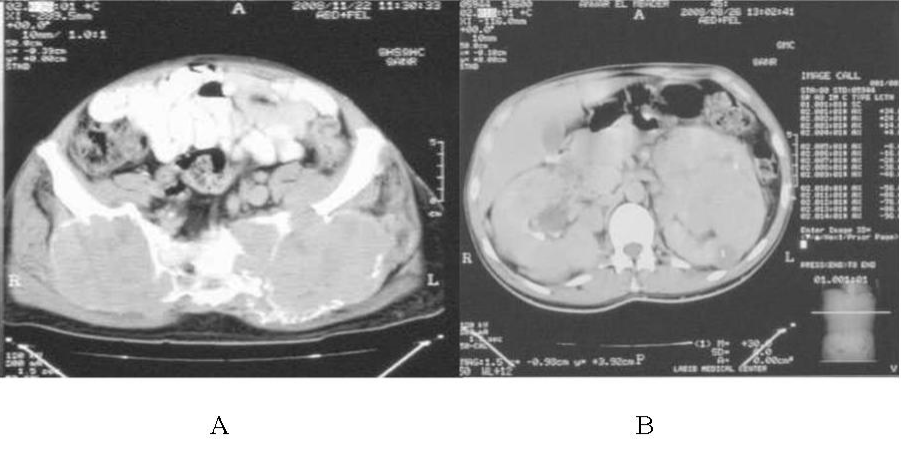
**(A) Axial MRI images showing bilateral sacroiliac heterogeneously enhancing masses**. (B) Abdominal CT scan showing large bilateral renal metastases.

In November 2007 and because of persistent low back pain, the patient underwent MRI of the spine and pelvis. These showed two large masses in the left and right sacroiliac and gluteus muscle regions measuring 11 × 9 cm and 8 × 7 cm, respectively, causing bone destruction of the left sacral wing with extension in the left sacral neural foramina (Figure 2A). Surgical biopsy was performed and this revealed metastatic atypical meningioma, with similar characteristics to its intracranial counterparts. Further work-up included CT scan of the abdomen which revealed large bilateral renal metastases (Figure 2B). CT of the chest was negative. The patient was evaluated by medical oncology but declined systemic chemotherapy. Brain MRI showed no disease progression. He is now receiving palliative care for pain control.

## Discussion

Meningioma is a common intracranial tumor that accounts for 14 to 19% of all primary intracranial neoplasms [1]. It is generally a benign tumor, but recurrence is not uncommon notably for atypical and anaplastic variants. A report by the World Health Organization indicated that 94.3% of meningiomas are benign with a 5-year recurrence rate of 3% compared to 38% and 78% for atypical and anaplastic meningiomas, respectively [2]. Distant metastases from benign meningiomas are extremely rare and almost all of the reported cases were associated with a large intracranial tumor [3]. On the other hand, the rate of distant metastases could reach 5% for atypical and 30% for anaplastic or malignant meningiomas [4].

Several histologic parameters have been identified as indicators of aggressive behavior and predictors of rapid recurrence of meningiomas. These include high cellularity, mitotic rate, nuclear pleomorphism, presence of foci of necrosis, and invasion of adjacent structures [5-9]. Other reported prognostic indicators of tumor recurrence include change in histologic morphology, malignant transformation, cellular heterogeneity, and multicentricity [10,11]. In the case presented here, atypical histologic features such as hypercellularity, relatively increased mitotic rate (4 mitoses per 10 high power fields), high proliferation index, and multicentricity were present.

According to one report, tumors that did metastasize usually had histologic features consistent with a malignant phenotype, such as focal necrosis, brain invasion, cellular pleomorphism, and frequent mitoses [12]. Meningiomas may disseminate through hematogenous, lymphatic, or cerebrospinal fluid routes [13]. The most common pathway of metastasis in meningiomas is considered to be through the cerebrospinal fluid but this does not explain extraneural metastases [14-16]. The other mode of metastatic spread occurs through the passage of tumor cells into venous channels and spread through the right blood circulation into the lungs, pleura, and other organs [17,18]. Isolated hepatic or renal metastasis may occur through the vertebral (meningorachidian) venous system[19]. These vertebral veins have several connections with the veins of the skull, spinal canal, vertebral column, and the intercostal veins of the thoracoabdominal wall.

The role of chemotherapy is limited in meningioma; there are no or limited proven benefit from any systemic therapy and no clear drug or combination regimen that has given consistent responses.

In order of descending frequency, metastases from meningiomas occur in the lungs and pleura, liver, lymph nodes, and bone [20]. Our patient had metastatic lesions in both sacroiliac regions and in both kidneys with remarkable symmetry in these locations. Sacral metastases from benign intracranial meningiomas are rare occurrences and could only be found in two reports [21,22]. However, renal metastases from meningiomas have been reported more often and constituted in one report 8% of all metastases from intracranial benign and malignant meningiomas [23]. We could not find any report with description of symmetrical features in meningioma metastases.

In conclusion, we report here the case of a 45-year-old man who developed bilateral sacroiliac and renal metastases from a recurrent intracranial meningioma of atypical histology 13 years after initial diagnosis.

## Consent

Written informed consent was obtained from the patient for publication of this case report and accompanying images. A copy of the written consent is available for review by the Editor-in-Chief of this journal.

## Competing interests

The authors declare that they have no competing interests.

## Authors' contributions

Authors' contributions: FG participated in paper editing. MA wrote the manuscript. MK and IAR reviewed the pathology. GH participated in paper editing. All authors read and approved the final manuscript.
